# Ultrasound-guided hydrodissection combined with radiofrequency for refractory occipital neuralgia: clinical outcomes, durability, and factors associated with repeat intervention

**DOI:** 10.3389/fneur.2026.1876927

**Published:** 2026-09-28

**Authors:** Xingchen Yao, Junpeng Liu, Baishan Wu, Xianglong Meng, Jincai Yang

**Affiliations:** 1 Department of Orthopaedic Surgery, Beijing Chaoyang Hospital, Capital Medical University, Beijing, China; 2 Department of Orthopaedic Surgery, Beijing Anzhen Hospital, Capital Medical University, Beijing, China; 3 Department of Pain Medicine, Beijing Chaoyang Hospital, Capital Medical University, Beijing, China

**Keywords:** greater occipital nerve entrapment, hydrodissection, radiofrequency treatment, repeat intervention, ultrasound guidance

## Abstract

**Objective:**

To evaluate clinical outcomes after ultrasound-guided hydrodissection combined with radiofrequency treatment for occipital neuralgia secondary to greater occipital nerve (GON) entrapment and to explore factors independently associated with repeat intervention.

**Methods:**

We retrospectively reviewed 81 patients with GON entrapment who underwent ultrasound-guided hydrodissection combined with radiofrequency treatment at Beijing Chaoyang Hospital between February 2024 and June 2025. The mean follow-up was 12.39 months. Pain intensity, headache-related disability, and depressive symptoms were assessed preoperatively and 3 months after treatment using the Visual Analogue Scale (VAS), Migraine Disability Assessment (MIDAS), and 10-item Center for Epidemiologic Studies Depression Scale (CESD-10), respectively. Kaplan–Meier analysis was used to estimate the median duration of symptom relief. Multivariable logistic regression was performed to identify factors independently associated with repeat intervention. Receiver operating characteristic analysis was used to determine an optimal cutoff, and subgroup analyses were conducted to assess the consistency of the association.

**Results:**

At 3 months, the mean VAS score decreased from 6.56 ± 1.63 preoperatively to 1.88 ± 1.05, MIDAS grades improved significantly, and the mean CESD-10 score decreased from 12.41 ± 7.26 to 7.63 ± 5.14 (all *p* < 0.05). The proportion of patients satisfied with treatment was 87.7%. One patient experienced local anesthetic systemic toxicity, corresponding to a complication rate of 1.23%. The median duration of symptom relief was 6 months. Multivariable logistic regression identified age as independently associated with repeat intervention (OR, 0.947; *p* = 0.022), with an optimal cutoff of 56.5 years. The inverse association between age and repeat intervention was generally consistent across prespecified subgroups, with no statistically significant interactions (all P for interaction > 0.05).

**Conclusion:**

In this retrospective single-arm cohort, ultrasound-guided hydrodissection combined with radiofrequency treatment was associated with improvements in pain, headache-related disability, and depressive symptoms, together with high patient satisfaction. Older age was independently associated with a lower likelihood of repeat intervention.

## Introduction

The global prevalence of neck pain and headache approaches 86.8% (1), with a rising socioeconomic burden (2). Greater occipital nerve (GON) entrapment is an important cause of occipital neuralgia. Abnormalities involving the posterior cervical muscles, fascial attachments, and soft-tissue adhesion or inflammation may contribute to nerve irritation or compression. Patients typically present with paroxysmal stabbing or electric shock-like pain in the occipital region, which may radiate to the vertex, temporal region, or even the periorbital area, substantially impairing sleep, work, and quality of life (3).

Initial management of occipital neuralgia caused by GON entrapment generally includes pharmacologic and physical therapies. However, patients with refractory disease who do not respond adequately to conservative treatment or cannot tolerate medication-related adverse effects may require an interventional procedure. GON decompression, corticosteroid or botulinum toxin injection, and occipital artery resection have demonstrated clinical benefit for occipital neuralgia associated with fascial entrapment. Nevertheless, each approach has limitations. Surgical decompression requires a relatively large incision and may be followed by prolonged recovery or local scarring, whereas injection therapy may provide only temporary relief. Minimally invasive interventions have therefore emerged as important alternatives. Ultrasound guidance offers real-time visualization, procedural precision, and minimal invasiveness. Hydrodissection can separate the entrapped GON from adjacent adherent tissues by injecting a solution around the nerve, potentially reducing mechanical compression. Adjunctive radiofrequency treatment of the surrounding fascia may further release tense fascial tissue and contribute to sustained symptom improvement. However, current evidence is derived largely from case reports and small retrospective series; short- to intermediate-term outcomes, durability, and patient-reported treatment experience have not been systematically evaluated (4).

In this retrospective study, we evaluated patients with occipital neuralgia caused by GON entrapment who underwent ultrasound-guided hydrodissection combined with radiofrequency fascial release. We assessed changes in pain, headache-related disability, depressive symptoms, treatment satisfaction, and duration of symptom relief. We also performed an exploratory analysis of factors associated with repeat intervention and examined whether these associations were consistent across clinically relevant subgroups. The findings were intended to provide preliminary evidence for optimizing the management of refractory GON entrapment-related occipital neuralgia.

## Materials and methods

### Study design and patient population

We retrospectively reviewed data from 81 patients admitted to Beijing Chaoyang Hospital, Capital Medical University, between February 2024 and June 2025 for ultrasound-guided hydrodissection combined with radiofrequency treatment of occipital neuralgia caused by GON entrapment. The study protocol was approved by the Ethics Committee of Beijing Chaoyang Hospital (approval no. 2024-ke-22) and was conducted in accordance with the Declaration of Helsinki. The ethics committee classified the study as a retrospective clinical audit and waived the requirement for written informed consent specifically for research participation. All patients provided standard written informed consent for the clinical intervention. Written informed consent was also obtained for publication of the case presentation and ultrasound images. Follow-up was performed by an independent assessor through March 2026. The minimum follow-up was 9 months, and the mean follow-up was 12.39 months.

### Eligibility criteria

The inclusion criteria were as follows: (1) occipital neuralgia diagnosed according to the International Classification of Headache Disorders, 3rd edition (ICHD-3); (2) refractory GON-related occipital neuralgia, defined as persistent or frequently recurrent pain that continued to substantially impair daily life despite at least 3 months of standardized conservative or pharmacologic therapy, trials of at least two drug classes with different mechanisms of action, and at least one interventional treatment (e.g., GON block) (5); (3) medication dependence or intolerance to pharmacologic therapy; (4) tenderness along the course of the GON, with typical pain reproduced by pressure over the semispinalis capitis on the symptomatic side (approximately 2 cm inferior to the external occipital protuberance and 2.5 cm lateral to the midline); (5) first treatment with the study procedure; and (6) complete follow-up data.

The exclusion criteria were as follows: (1) active infection at the puncture site; (2) coagulopathy or ongoing anticoagulant/antiplatelet therapy that could not be discontinued as required; (3) severe psychiatric illness, cognitive impairment, or inability to understand and provide informed consent; (4) allergy to a local anesthetic or other injectate component; (5) secondary occipital neuralgia caused by a defined structural lesion, including a cervical tumor, infection, fracture/dislocation, or C1-C2 arthropathy; (6) coexisting migraine, cluster headache, chronic tension-type headache, or another headache disorder; (7) pregnancy or lactation; (8) an implanted pacemaker or other electronic device because of the potential for radiofrequency interference; and (9) physical therapy, botulinum toxin type A injection, acupuncture, ozone therapy, or another treatment within the preceding 3 months that could confound outcome assessment.

A total of 81 patients met the eligibility criteria and were included in the analysis (Figure 1).

**Figure 1:**
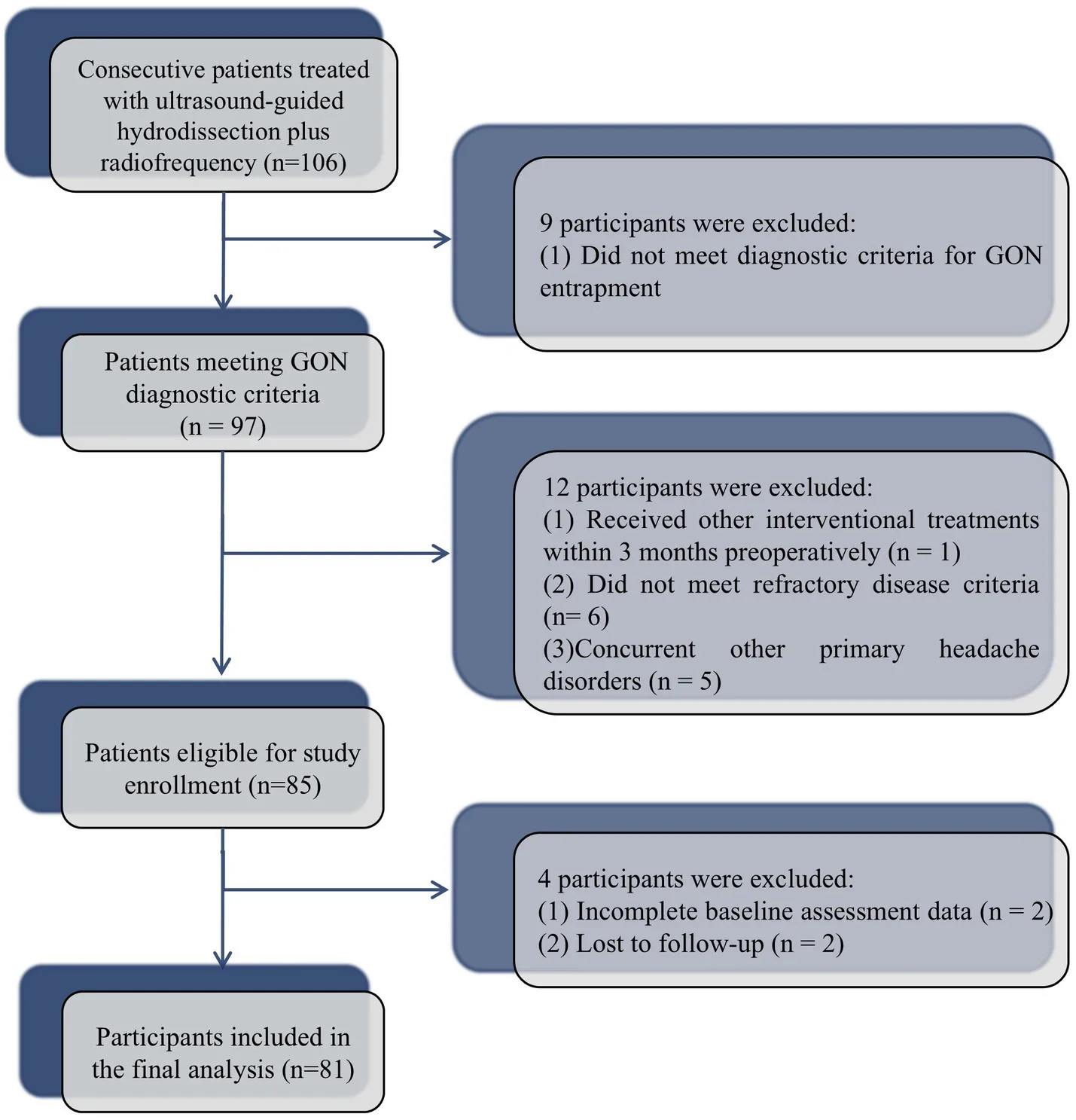
Flow diagram of patient selection. GON, greater occipital nerve.

### Medications and equipment

The hydrodissection solution was prepared by adding one 1-mL ampule of compound betamethasone injection (Organon, Shanghai, China; containing 5 mg betamethasone dipropionate and 2 mg betamethasone sodium phosphate) and one 5-mL ampule of 2% lidocaine hydrochloride (Otsuka Pharmaceutical Co., Ltd., Tianjin, China; 100 mg lidocaine, 20 mg/mL) to normal saline to obtain a total volume of 50 mL. The resulting theoretical lidocaine concentration was approximately 2 mg/mL.

A low-temperature plasma radiofrequency system (LZ-GP-300; Zhejiang Luangzi Medical Equipment, Hangzhou, China) was used. A disposable bipolar radiofrequency electrode (model GB4824) with a 0.9-mm-diameter, 110-mm-long working tip was employed. The system was operated in cutting mode at level 2 and coagulation mode at level 1. Under a standard 200-ohm load, the cutting-mode output was 100 W; the intraoperative coagulation output was 70 W, and the operating frequency was 1.71 MHz. The low-temperature radiofrequency energy typically ranged from 40 to 70 °C. Tissue thermal effects were controlled through standardized power settings to promote procedural consistency.

### Periprocedural management and treatment protocol

Routine imaging and preprocedural assessments were completed, and hair below the superior nuchal line was clipped when necessary. The patient was placed prone with the head slightly flexed. After povidone-iodine skin preparation and sterile draping, a low-frequency curvilinear transducer was used to obtain the standard semispinalis capitis view. The skull base, splenius capitis, semispinalis capitis, obliquus capitis inferior, and other relevant structures were identified. The GON was typically visualized between the semispinalis capitis and obliquus capitis inferior muscles (Figure 2).

**Figure 2:**
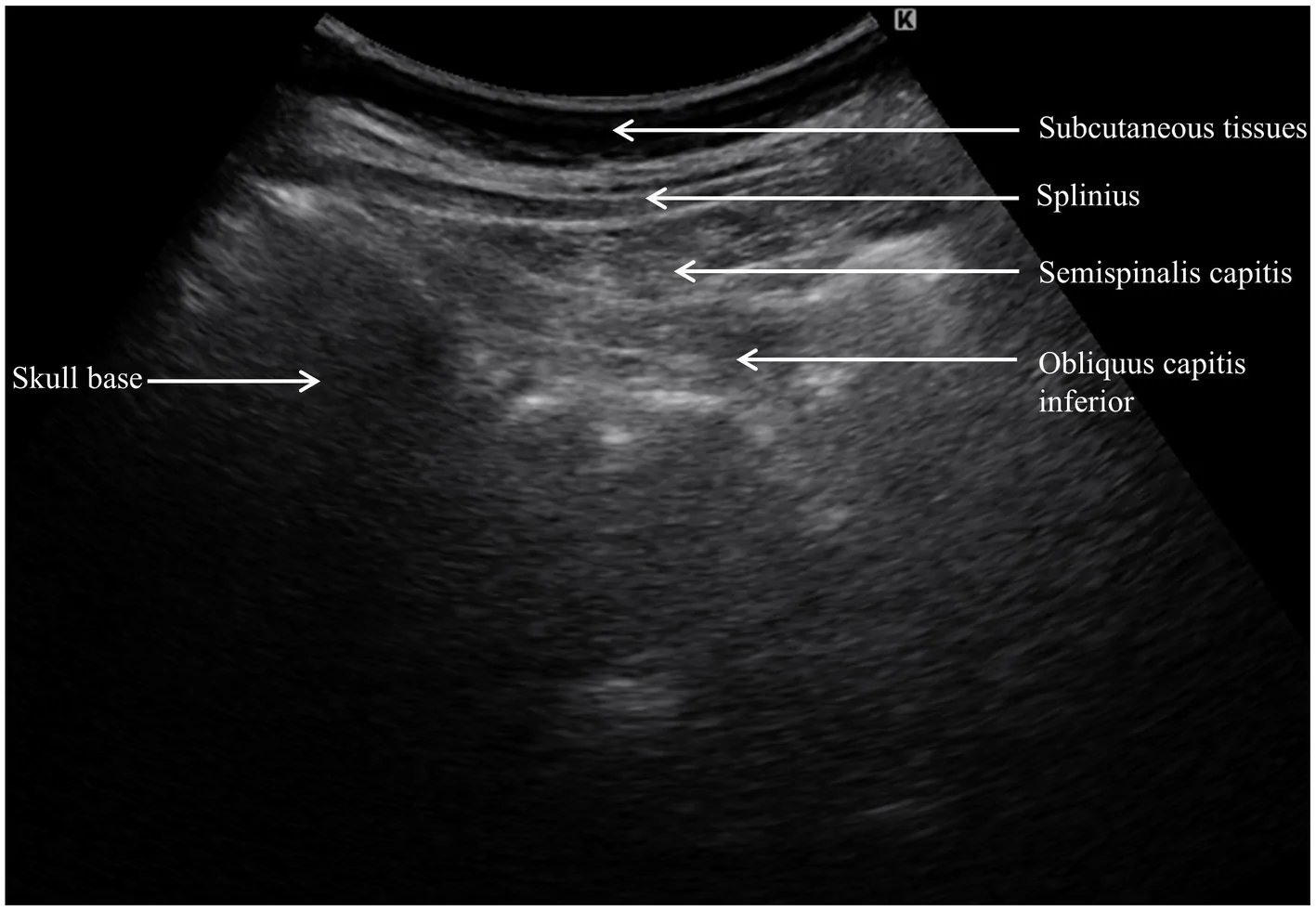
Ultrasound anatomy along the course of the greater occipital nerve (45-degree oblique scan at the C2 spinous-process level). Ultrasound clearly demonstrates the posterior occipital layers from superficial to deep, including the subcutaneous tissue, splenius, semispinalis capitis, obliquus capitis inferior, and skull base. This image represents the standard localization view obtained before ultrasound-guided treatment of GON entrapment. GON, Greater occipital nerve.

After local infiltration with a small volume of 1% lidocaine diluted 1:1 with normal saline, the needle was advanced using an entirely in-plane approach under continuous ultrasound visualization to the plane between the semispinalis capitis and obliquus capitis inferior muscles, with care taken to avoid the occipital artery. The prepared solution was then injected slowly under real-time ultrasound guidance to separate the nerve from the surrounding adherent tissue and create a clearly visible anechoic fluid plane (6). The procedure is demonstrated in Supplementary Video 1. Approximately 15 mL was administered on each side (30 mL in total for bilateral treatment). After 5 min, sensory blockade was assessed by applying a pinprick at a site distant from the injection point within the GON distribution. Absence of pinprick pain in the target territory was considered evidence of a successful block and provided additional confirmation of appropriate targeting.

After hydrodissection, a low-temperature plasma radiofrequency electrode was used for fascial release. Two sites were treated on each side: the fascial tissues adjacent to the obliquus capitis inferior and semispinalis capitis. Radiofrequency energy was applied for 3–5 s at each site, with complete visible fascial release used as the procedural endpoint. A minimum distance of 5–8 mm was maintained between the active electrode tip and the anatomical course of the GON to avoid direct contact with or thermal injury to the nerve. The procedure is demonstrated in Supplementary Video 2. Pain responses and limb movement were monitored throughout the intervention for signs of neurologic injury. After treatment, the electrode was withdrawn slowly. Hemostasis was achieved with local pressure, and a sterile dressing was applied. Patients were observed in the treatment area for 30 min, advised to avoid strenuous neck movement on the day of treatment, and monitored for puncture-site bleeding, infection, and other adverse events.

### Clinical outcomes

Baseline characteristics, disease-related information, and preoperative medication use were recorded. Complications, symptom recurrence, duration of symptom relief, and repeat interventions were documented throughout follow-up. Duration of symptom relief was defined as the interval from completion of the index radiofrequency procedure to pain recurrence or any repeat intervention for occipital neuralgia. Recurrence was considered to have occurred when the VAS score returned to the preoperative level, the patient reported recurrent pain, or the degree of relief was no longer satisfactory (7). A repeat-intervention event was defined as the actual receipt of an additional targeted treatment after recurrence or persistence of relevant symptoms and clinical reassessment. Events included procedures performed at the study institution and procedures performed elsewhere for personal reasons when the patient remained in follow-up at the study institution and had reported satisfaction with the index treatment (8). No patient underwent a third intervention during the mean follow-up of 12.39 months.

Pain intensity was assessed preoperatively and at 3 months using the Visual Analogue Scale (VAS). Headache-related disability over the preceding 3 months was assessed using the five-item Migraine Disability Assessment (MIDAS) questionnaire (9, 10). The questionnaire quantifies days of missed work or school, days with productivity reduced by at least 50% at work or school, days of missed household work, days with productivity reduced by at least 50% in household work, and days of missed family, social, or leisure activities. Total days are summed and categorized into four grades: grade I, 0–5 (little or no disability); grade II, 6–10 (mild disability); grade III, 11–20 (moderate disability); and grade IV, ≥21 (severe disability). Depressive symptoms were assessed using the 10-item Center for Epidemiologic Studies Depression Scale (CESD-10; score range, 0–30; Cronbach alpha = 0.815) (11), with scores ≥10 indicating an increased risk of depression (12). Patient satisfaction was measured using the Patient Satisfaction Index (PSI) from the North American Spine Society outcome questionnaire (13). PSI grades I and II were classified as satisfied, whereas grades III and IV were classified as dissatisfied.

### Statistical analysis

Statistical analyses were performed using IBM SPSS Statistics, version 25.0 (IBM Corp., Armonk, NY, United States). The Kolmogorov–Smirnov test was used to assess distributional normality. Categorical variables are presented as frequencies and percentages, and normally distributed continuous variables as means ± standard deviations. Preoperative and postoperative continuous outcomes were compared using paired-samples t tests when normally distributed. Binary paired outcomes were compared using the McNemar test, and ordinal paired outcomes using the Wilcoxon signed-rank test. All tests were two-sided, and *p* < 0.05 was considered statistically significant. Kaplan–Meier analysis was performed with duration of symptom relief as the time variable and symptom recurrence as the event to estimate the median time to recurrence.

Repeat intervention was additionally examined as an exploratory clinical outcome (14, 15). This outcome was intended to capture patients’ overall appraisal of the index treatment (16). Pre-specified candidate variables, including age, sex, preoperative symptom duration, pain laterality, preoperative VAS score, and duration of symptom relief, were first screened using univariable logistic regression. Due to marked multicollinearity among subjective efficacy-assessment scores, only the VAS score was incorporated into the regression model. Categorical variables were dummy coded, and the enter method was used. Odds ratio (OR), 95% confidence interval (CI), and exact *p* values were reported. Given the exploratory nature of the analysis, variables with *p* < 0.10 in univariable analysis were entered into the multivariable logistic regression model.

The events-per-variable ratio was calculated to assess sample adequacy. Multicollinearity was evaluated using variance inflation factors (VIF) and tolerance values. Influential observations were assessed using Cook’s distance and DFBETAs. Overall model significance was evaluated using the omnibus likelihood-ratio test; calibration was assessed using the Hosmer-Lemeshow test; and explanatory performance was summarized using the Cox-Snell and Nagelkerke R^2^ statistics. Variables with *p* < 0.05 in the multivariable model were considered independently associated with repeat intervention.

RStudio version 4.2.1 (R Foundation for Statistical Computing, Vienna, Austria) was used to generate receiver operating characteristic (ROC) curves for independently associated factors and calculate the area under the curve (AUC). Internal validation with 1,000 bootstrap resamples was used to reduce optimism arising from single-cohort model development, generate a calibration curve, and quantify prediction error. For continuous factors, the optimal cutoff was identified by maximizing the Youden index; sensitivity and specificity were corrected using 1,000 bootstrap resamples.

For subgroup analysis, the continuous factor independently associated with repeat intervention was dichotomized at its median. Analyses were performed using the jstable and forestploter packages in RStudio version 4.2.1, and results were displayed in a forest plot. Effect modification was assessed using interaction terms; P for interaction < 0.05 was considered statistically significant. These analyses explored whether the association between the identified factor and repeat intervention differed across subgroups.

## Results

### Patient characteristics

The study included 81 patients with occipital neuralgia who underwent ultrasound-guided hydrodissection combined with radiofrequency treatment (Table 1). The mean age was 58.36 years, and the mean preoperative symptom duration was 50.40 months. Of the patients, 57 (70.4%) were women and 24 (29.6%) were men. Bilateral pain was present in 71 patients (87.7%). Three patients did not meet the prespecified recurrence criteria during follow-up. Kaplan–Meier analysis estimated a median duration of symptom relief of 6 months (95% CI, 5.770–6.230). Overall, 71 of 81 patients (87.7%) reported satisfaction with treatment (PSI grades I or II), and 30 patients underwent a repeat intervention during follow-up. One patient experienced treatment-related local anesthetic systemic toxicity (see Figure 3).

**Table 1:** Patient characteristics (*n* = 81).

Variable	Overall (*n* = 81)
Age, years	58.36 ± 11.54
Preoperative symptom duration, months	50.40 ± 84.50
Sex, *n* (%)	
Male	24 (29.6%)
Female	57 (70.4%)
Pain laterality, *n* (%)	
Unilateral	10 (12.3%)
Bilateral	71 (87.7%)
Duration of symptom relief, months	6.11 ± 1.38
Follow-up duration, months	12.39 ± 2.42
Repeat intervention, *n* (%)	
Yes	30 (37.0%)
No	51 (63.0%)
Patient Satisfaction Index, *n* (%)	
I	47 (58.0%)
II	24 (29.6%)
III	9 (11.1%)
IV	1 (1.2%)
Complications, *n* (%)	1 (1.23%)
Medication use during the preceding 3 months, *n* (%)	
Nonsteroidal anti-inflammatory drugs/acetaminophen	68 (84.0%)
Gabapentin or pregabalin	56 (69.1%)
Carbamazepine or oxcarbazepine	29 (35.8%)
Neurotrophic agents	18 (22.2%)
Muscle relaxants	31 (38.3%)
Tricyclic antidepressants	21 (25.9%)

Data are presented as $\bar{x}$ ± s or *n* (%).

**Figure 3:**
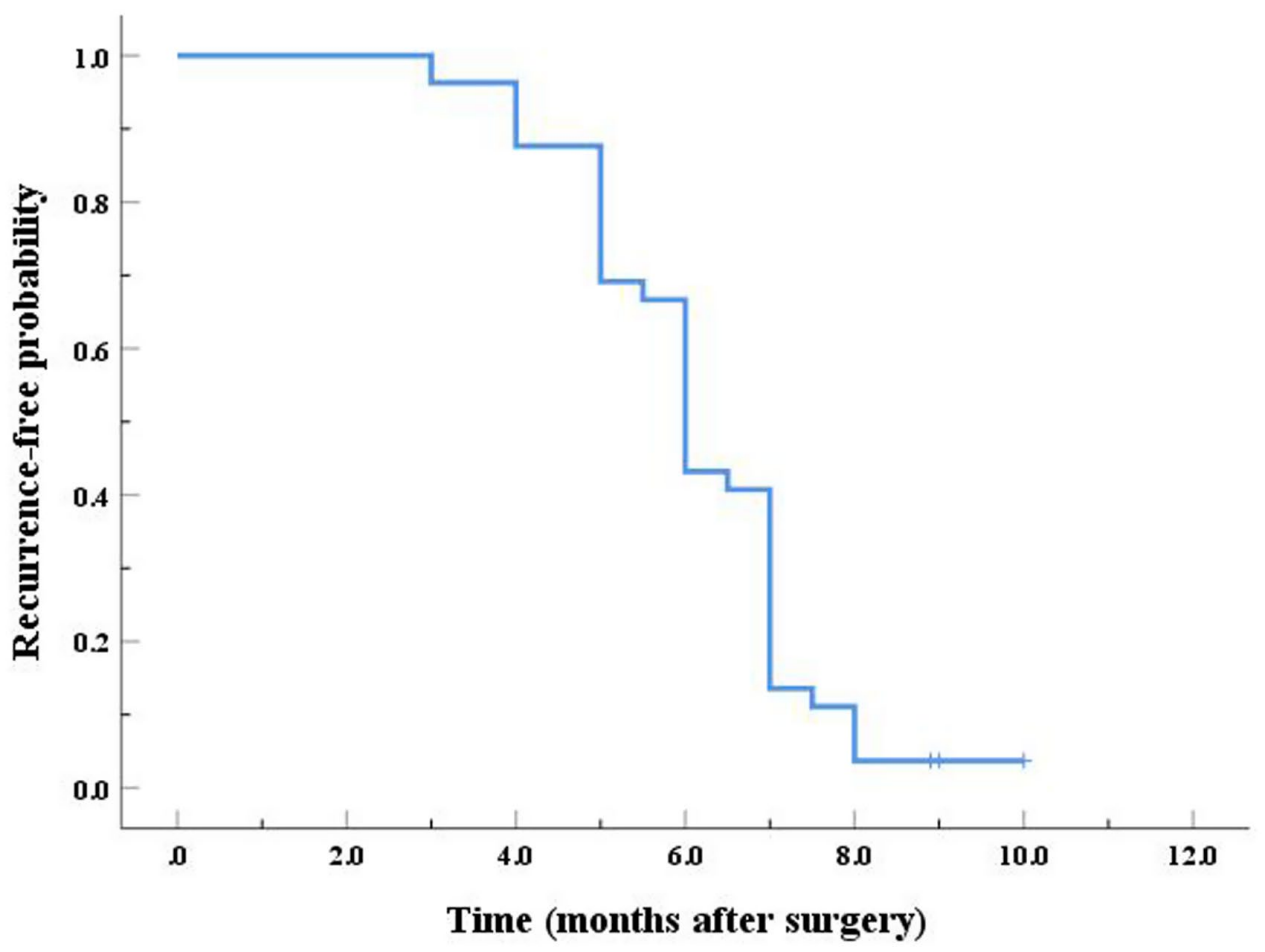
Kaplan–Meier curve for duration of symptom relief.

### Clinical outcomes

Compared with baseline, the mean VAS score decreased from 6.56 to 1.88 at 3 months. MIDAS scores and grades also improved significantly. The mean CESD-10 score decreased from 12.41 to 7.63, and the proportion of patients with CESD-10 scores ≥10 decreased from 51.9% (42/81) to 33.3% (27/81). All assessed outcomes showed statistically significant improvement at 3 months (all *p* < 0.05; Table 2).

**Table 2:** Comparison of preoperative and 3-month outcomes (*n* = 81).

Variable	Preoperative	3 months after treatment	*p* value
VAS score	6.56 ± 1.63	1.88 ± 1.05	< 0.001
MIDAS score	19.12 ± 6.74	6.47 ± 4.21	< 0.001
MIDAS grade, *n* (%)			< 0.001
I	0	55 (67.9%)	
II	9 (11.1%)	23 (28.4%)	
III	33 (40.7%)	3 (3.7%)	
IV	39 (48.1%)	0	
CESD-10 score	12.41 ± 7.26	7.63 ± 5.14	< 0.001
CESD-10 category, *n* (%)			< 0.001
< 10	39 (48.1%)	54 (66.7%)	
≥10	42 (51.9%)	27 (33.3%)	

Data are presented as $\;\bar{x}$ ± s or *n* (%). VAS, Visual Analogue Scale; MIDAS, Migraine Disability Assessment; CESD-10, 10-item Center for Epidemiologic Studies Depression Scale.

### Logistic regression analysis

Repeat intervention was the outcome of interest and occurred in 30 of 81 patients. Univariable analysis identified age (OR, 0.938; 95% CI, 0.896–0.982; *p* = 0.006) and *preoperative* symptom duration (OR, 0.992; 95% CI, 0.984–1.001; *p* = 0.066) as candidate factors *at* the prespecified *p* < 0.10 threshold. Sex, pain laterality, duration of symptom relief, and preoperative VAS score were not statistically significant (Table 3).

**Table 3:** Univariable logistic regression analysis of repeat intervention.

Variable	OR	95% CI	*P*
Age	0.938	(0.896, 0.982)	0.006
Preoperative symptom duration	0.992	(0.984, 1.001)	0.066
Sex (reference: female)	0.609	(0.218, 1.700)	0.343
Pain laterality (reference: unilateral)	0.698	(0.166, 2.933)	0.624
Duration of symptom relief	1.132	(0.811, 1.580)	0.466
Preoperative VAS score	1.091	(0.827, 1.438)	0.539

OR, Odds Ratio; CI, Confidence Interval; VAS, Visual Analogue Scale.

Age and preoperative symptom duration were entered simultaneously into a multivariable binary logistic regression model using the enter method. With 30 events and two predictors, the events-per-variable ratio was 15, satisfying the conventional threshold of at least 10. Both predictors were continuous. The tolerance was 0.908 and the VIF was 1.101, indicating no meaningful multicollinearity. All observations were retained. The maximum Cook’s distance was 0.465, with all values <0.5; assessment of DFBETAs likewise identified no highly influential observations. In the final model, age was independently associated with repeat intervention (OR per year, 0.947; 95% CI, 0.904–0.992; *p* = 0.022), indicating lower odds of repeat intervention with increasing age. Preoperative symptom duration was not independently associated with the outcome (OR per month, 0.994; 95% CI, 0.986–1.003; *p* = 0.199).

The omnibus test was statistically significant (χ^2^ = 10.780, df = 2, *p* = 0.005). The Cox-Snell R^2^ was 0.125 and the Nagelkerke R^2^ was 0.170. The *Hosmer-Lemeshow* goodness-of-fit test suggested acceptable calibration (χ^2^ = 7.372, df = 8, *p* = 0.497). The overall classification accuracy was 67.9%.

An ROC curve was generated for age (Figure 4A), yielding an AUC of 0.696 (95% CI, 0.575–0.817). The cutoff maximizing the Youden index was 56.5 years, with a sensitivity of 0.633 and specificity of 0.745. After correction with 1,000 bootstrap resamples, sensitivity was 0.667 and specificity was 0.740. A calibration curve for the age-only model was generated to compare predicted probabilities with observed repeat-intervention rates; the 45-degree line represented perfect calibration (Figure 4B). The mean absolute error was 0.035, the mean squared error was 0.00175, and the 90th percentile of the absolute error was 0.074, indicating generally good apparent agreement.

**Figure 4:**
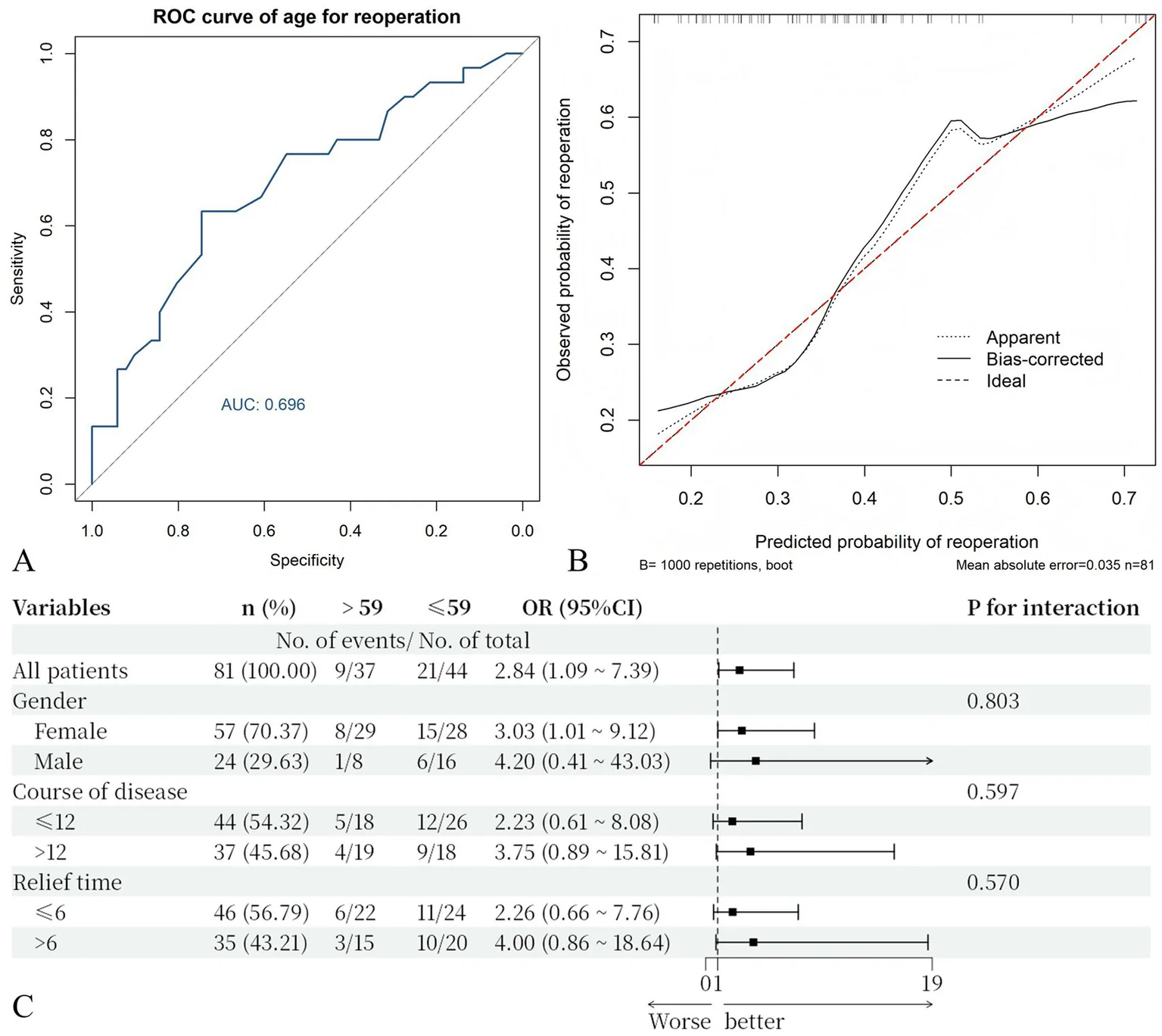
Receiver operating characteristic curve, calibration curve, and subgroup analysis for age in relation to repeat intervention. **(A)** ROC curve for age as a discriminator of repeat intervention; AUC = 0.696. **(B)** Calibration curve based on 1,000 bootstrap resamples. The x-axis shows the model-predicted probability of repeat intervention and the y-axis the observed probability. The gray dotted line represents apparent calibration, the black solid line the bootstrap bias-corrected calibration, and the red dashed line ideal calibration. **(C)** Forest plot of the association between age and repeat intervention across subgroups. Age was dichotomized at the median of 59 years, with >59 years as the reference group. Results are stratified by symptom duration, duration of symptom relief, and sex. For each subgroup, the plot presents the proportion of the cohort, number of events/total participants in each age category, OR with 95% CI, and P for interaction. ROC, receiver operating characteristic; AUC, area under the curve; OR, odds ratio; CI, confidence interval.

### Subgroup analysis

We performed subgroup analysis using the median age (59 years), median disease duration (12 months), and median duration of pain relief (6 months) as stratification cut-off values, with sex included as stratifying factor, to assess the consistency of the association between age and repeat intervention (Figure 4C). The negative correlation between increasing age and decreasing willingness to undergo reoperation showed consistent effect directions in all subgroups, suggesting that this association has good stability (all P for interaction > 0.05).

### Case presentation

A 64-year-old female presented with intermittent pricking pain in the right occipital region without obvious inducement 5 years ago. The pain radiated from the posterior neck to the top of the head, right orbit, and temporal region. Each attack lasted 1–2 days, with 6–8 attacks half a year; the pain could be induced by desk work, head-down movements, and pressing. She had received oral non-steroidal anti-inflammatory drugs, pregabalin and other medications successively, but the pain control was poor. Three months before admission, the pain severity and attack frequency progressed, and conservative intervention was ineffective, seriously affecting the patient’s sleep and daily life. The patient was in good health in the past, without a history of cervical trauma or surgery. Physical examination showed clear consciousness, no abnormalities in cranial nerve examination, good cervical mobility without limitation, no abnormalities in muscle strength, sensation, and reflex of both upper limbs, and no local skin infection or rash. Definite tender points could be palpated in the course of the right greater occipital nerve (2 cm below the external occipital protuberance and about 2.5 cm lateral to the midline) and the space between the semispinalis capitis and obliquus capitis inferior muscles at the C2 level; pressing could reproduce the typical radiating pain in the right occipital region. Auxiliary examinations showed no relevant abnormalities in cervical and cranial MRI.

After completing preoperative relevant examinations, the patient underwent ultrasound-guided hydrodissection combined with radiofrequency therapy for right greater occipital nerve entrapment. Three months after surgery, the patient’s pain was significantly relieved: VAS score decreased from 7 points at baseline to 2 points, MIDAS grade decreased from grade IV to grade II, and CESD-10 score decreased from 15 points to 8 points. The patient was satisfied with the treatment effect, and her confidence in treatment was significantly improved. Seven months after surgery, the frequency and severity of pain attacks returned to a level similar to that before surgery, and the patient received reoperation, which also achieved satisfactory curative effect.

### Adverse event case presentation

One patient developed manifestations consistent with local anesthetic systemic toxicity. This 63-year-old woman received injections at multiple sites: in addition to the perineural GON intervention, she underwent local injection for concomitant trapezius myofascitis. The cumulative volume of 2% lidocaine was 15 mL, corresponding to a total dose of 300 mg; her body weight was 72 kg. Approximately 5 min after completion of the injections, during the postprocedure observation period, she developed sudden dizziness, palpitations, dysarthria, and limb weakness. No seizure, loss of consciousness, dyspnea, severe arrhythmia, or other major reaction occurred. The procedure was stopped immediately. She was placed in the Trendelenburg position and received electrocardiographic and oxygen-saturation monitoring, supplemental oxygen through a nasal cannula, intravenous fluids, and supportive measures to maintain airway patency and hemodynamic stability. All symptoms resolved gradually within 20 min with conservative management. No specific resuscitative therapy was required, and no sequelae were observed.

## Discussion

The GON is a sensory nerve that transmits nociceptive and other sensory signals to the dorsal horn. Chemical irritation, such as exposure to inflammatory mediators, or mechanical irritation from scar tissue affecting the nerve or its tissue bed may produce pain and tenderness in the suboccipital region (17). In this cohort of patients with refractory GON entrapment-related occipital neuralgia, ultrasound-guided hydrodissection combined with radiofrequency treatment was associated with substantial short-term symptom improvement. At 3 months, the mean VAS score decreased from 6.56 ± 1.63 to 1.88 ± 1.05. Headache-related disability also improved: the proportion of patients with MIDAS grade IV decreased from 48.1 to 0%, whereas 67.9% were classified as grade I (18). The proportion with CESD-10 scores decreased from 12.41 to 7.63. The largest item-level contributions to the change in total CESD-10 score were observed for restless sleep, feeling that everything was an effort, and difficulty getting going (19).

The improvement in pain observed after treatment may reflect several components of the combined intervention. Ultrasound-guided hydrodissection permits precise delivery of the injectate into the potential space surrounding the GON and creates a visible fluid plane under real-time imaging. This may mechanically separate the nerve from adherent soft tissue and fascia and thereby reduce local irritation. The local anesthetic and corticosteroid components may provide short-term analgesia by inhibiting neural conduction and attenuating local inflammation, respectively. Radiofrequency treatment of the deep fascia of the semispinalis capitis may additionally reduce fascial tension and persistent mechanical irritation around the nerve, potentially contributing to sustained symptom improvement. The median duration of symptom relief in this cohort was 6 months. Conceptually, hydrodissection may provide prompt decompression, whereas radiofrequency fascial release may contribute to durability, making the combination capable of addressing both rapid pain relief and maintenance of benefit (17). However, fascial tension, local microcirculation, neural recovery, and tissue repair were not measured. The present data therefore cannot establish that radiofrequency treatment prolonged symptom relief through any specific biological mechanism. Moreover, because all patients received the same combination and no hydrodissection-only or non-radiofrequency control group was included, the independent or synergistic contribution of each component cannot be determined. These mechanistic interpretations remain hypotheses derived from prior research and require evaluation in prospective randomized controlled studies. Compared with landmark-guided puncture, ultrasound guidance improves visualization of nerves, fascial planes, and adjacent vessels and may therefore enhance targeting and reduce the risk of injury to neighboring structures (20). Only one mild episode of local anesthetic systemic toxicity occurred (21). This case highlights that even with standardized ultrasound-guided puncture and appropriately dosed local anesthesia, simultaneous multi-site puncture and multi-point infiltration may raise the risk of cumulative local anesthetic intoxication. Hence, the safety of ultrasound-guided radiofrequency therapy is not absolute; rigorous procedural standardization and individualized dose titration represent key prerequisites for perioperative safety. Clinically, low-dose anesthesia tailored to patient weight and lesion features, together with continuous intraoperative monitoring of vital signs and consciousness, enables early recognition and prompt management of adverse events and improves procedural safety and controllability.

Older age was independently associated with lower odds of repeat intervention. The age cutoff maximizing the Youden index was 56.5 years, and patients aged ≥57 years had fewer repeat-intervention events. The association was broadly consistent across the examined subgroups. These findings should be interpreted cautiously because they arose from an exploratory analysis of a small, single-center cohort and may not be generalizable. The relationship between age and repeat intervention may reflect age-related physiological, psychological, or contextual differences. Previous research suggests that older adults may have greater concerns about procedural safety and that pain thresholds may increase with age because of changes in peripheral nociceptive fibers, central nociceptive processing, and cortical activation (22). In contrast, younger and middle-aged adults may prioritize restoration of daily function and therefore be more likely to seek another intervention after pain recurrence. Importantly, the observed association may also be confounded by recurrence severity, financial circumstances, access to care, risk perception, comorbidities, and treatment satisfaction. The current evidence does not support age-based treatment or follow-up strategies; larger multicenter prospective studies are required to determine whether age has independent clinical relevance.

This study provides preliminary information on short-term outcomes and safety after ultrasound-guided hydrodissection combined with radiofrequency treatment for refractory occipital neuralgia and identifies age as a potential factor associated with repeat intervention. Several limitations should be acknowledged. First, the single-center retrospective design limits generalizability and is vulnerable to selection bias and residual confounding. Second, receipt of repeat intervention is influenced not only by symptoms but also by patient preferences, psychological state, financial circumstances, access to care, and clinician recommendations. Third, symptom improvement may have been affected by the natural fluctuation of pain, regression to the mean, placebo and expectation effects, concomitant pharmacologic or rehabilitative treatment, and unmeasured confounders. Fourth, without a parallel control group, the observed pre-post improvements cannot be interpreted as causal treatment effects. Because a fixed combination regimen was used, the relative contribution of hydrodissection, lidocaine, corticosteroid, and radiofrequency treatment cannot be determined. The study also did not systematically compare this approach with other interventions. Multicenter studies with larger samples, longer follow-up, appropriate comparator groups, and, where feasible, factorial or randomized designs are needed to clarify durability, safety, and comparative effectiveness (17).

## Conclusion

In this retrospective single-arm cohort, ultrasound-guided hydrodissection combined with radiofrequency treatment for refractory occipital neuralgia was associated with improvements in pain and related clinical outcomes. Older age was independently associated with fewer repeat-intervention events, and no statistically significant effect modification was detected across the examined subgroups.

## Data Availability

The raw data supporting the conclusions of this article will be made available by the authors, without undue reservation.
